# Identification of Candidate Genes Involved in the Determinism of Pollen Grain Aperture Morphology by Comparative Transcriptome Analysis in Papaveraceae

**DOI:** 10.3390/plants12071570

**Published:** 2023-04-06

**Authors:** Ismael Mazuecos-Aguilera, Víctor N. Suárez-Santiago

**Affiliations:** Department of Botany, Faculty of Sciences, University of Granada, 18071 Granada, Spain; ismaag@ugr.es

**Keywords:** genetic determinism, Papaveraceae, pollen aperture, transcriptome analysis, RNA-seq

## Abstract

In the last decade, certain genes involved in pollen aperture formation have been discovered. However, those involved in pollen aperture shape remain largely unknown. In *Arabidopsis*, the interaction during the tetrad development stage of one member of the ELMOD protein family, ELMOD_E, with two others, MCR/ELMOD_B and ELMOD_A, can change the morphology of apertures from colpus (elongated) to pore (round). Here, comparative transcriptome analysis is used to identify candidate genes involved in the determination of pollen aperture morphology in Papaveraceae (order Ranunculales). Furthermore, the role of ELMOD genes in the genetic determinism of aperture shape was tested by comparative analysis of their expression levels using RNA-seq data and RT-qPCR. Two pairs of species belonging to two different subfamilies were used. Within each pair, one species has colpate pollen and the other porate (Fumarioideae—*Dactylicapnos torulosa*, 6-colpate, and *Fumaria bracteosa*, pantoporate; Papaveroideae—*Eschsholzia californica*, 5–7 colpate, and *Roemeria refracta*, 6-porate). The transcriptomes were obtained at the tetrad stage of pollen development. A total of 531 DEGs were found between the colpate and porate pollen species groups. The results from RNA-seq and RT-qPCR indicate that pollen aperture shape is not determined by the relative expression levels of *ELMOD* family genes in Papaveraceae. However, genes related to callose wall formation or cytoskeleton organisation were found, these processes being involved in pollen aperture formation. In addition, transcriptomes from anthers with pollen during the tetrad stage of three species (*D. torulosa*, *R. refracta*, and *F. bracteosa*) were obtained for the first time. These data will be available for further studies in the field of floral evolution and development.

## 1. Introduction

Pollen apertures, sites with little or no exine (the name for the outermost wall of the pollen), represent some of the most characteristic and well-defined elements of the pollen surface [1,2]. The apertural pattern of the pollen grain is defined by the shape, number, and position (polar or equatorial) of their apertures, and changes in this apertural pattern have been related to evolutionary advantages or variations in the efficiency of its functions. Thus, for example, the transition from pollen with one polar aperture (typical of basal angiosperms and monocots) to pollen with three apertures in equatorial positions has been interpreted as a key innovation involved in the success and diversification of eudicots [1].

Regarding shape, Spermatophyte pollen shows two predominant morphotypes of apertures: elongated, furrow-like apertures (colpus and sulcus) and round, pore-like apertures (porus and ulcus) [3]. Aperture morphology remains stable at the intraspecific level, although some species have apertures that combine both types, with an elongated ectoaperture and a rounded endoaperture (colporus). Species with furrow-like apertures are more numerous than those with porate pollen [1,4]. The significance of variation in aperture shape between taxa is poorly understood, although a growing consensus holds that it is related to environmental xericity. One of the functions attributed to apertures is to allow the rigid exine to adjust to changes in pollen volume due to dehydration/rehydration during pollination, the harmomegathy process [5,6,7]. In response to dehydration, the membrane of the aperture sites folds inward, so that the edges of each aperture touch each other, closing up the aperture site [8]. Božič and Šiber [9], in their study of the modelling of pollen infolding, showed that pollens with elongated apertures achieved successful closure with little stretching, and that this process occurred gradually, allowing response to slight moisture changes in the environment. By contrast, pore-like aperturates cannot adequately seal the aperture and undergo major stretching at the margins of the aperture. In addition, if a porate pollen grain greatly reduces its volume, it adopts a mirror buckling geometry with a considerable amount of stretching energy concentrated at the edges. This change in geometry requires a transition from a high-energy state to bistability, which would not allow response to minor changes in moisture [10,11].

However, porate pollen is distributed throughout most of the angiosperm tree, although usually restricted to a few species in a given group, and is seldom fixed at large taxonomic scales [12], and many of these species inhabit xeric environments. It has been observed that, unlike most pollen, many porate pollen grains disperse when only partially dehydrated, giving them a faster germination rate than fully dehydrated pollens [13]. Prieu et al. [12], on the basis of this evidence, explained why porate pollens have evolved many times during angiosperm history but have been less successful than furrow pollen. According to these authors, faster germination could constitute a competitive advantage of porate pollen grains, which would be selected in the short term. However, their lower tolerance to dehydration would lead to their elimination in the long term. The independent evolution of porate pollen among the different groups of angiosperms suggests a common molecular mechanism for its emergence.

In the last decade, some genes involved in the formation of pollen apertures have been discovered (*INAPERTURATE POLLEN 1*, *INP1* [14]; *D6 PROTEIN KINASE-LIKE3*, *D6PKL3* [15]; *DEFECTIVE IN APERTURE FORMATION1*, *OsDAF1* [16]; *INAPERTURATE POLLEN2*, *INP2* [17]). However, only *INP1* has been shown to affect apertural morphology by decreasing colpus length when the concentration of INP1 protein is reduced [14]. Recently, a protein family (ELMOD; engulfment and cell motility domain containing) has been found in *Arabidopsis* that acts upstream of the aperture formation pathway, in which the interaction of one member, ELMOD_E, with two others, MACARON(MCR)/ELMOD_B and ELMOD_A, can change the morphology of apertures from colpus to pore [18]. MCR and ELMOD_A are paralogues with redundancy in function (although MCR shows dominance) that participate in aperture domain specification by affecting the number of domains (dose dependent), but not the morphology of the apertures. *MCR* and *ELMOD_A* are both expressed at or near the tetrad stage of pollen development [18]. The neomorphic activity of ELMOD_E occurs only when gene expression levels are high during the tetrad phase, as when its expression is induced with the MCR promoter. Experiments by Zhou et al. [18] show that high levels of MCR counteract the neomorphic activity of ELMOD_E, suggesting that both proteins compete for the same interactors. In *Arabidopsis*, a colpate species, *ELMOD_E* expression during tetrads is low and thus does not influence the aperture pattern. In contrast to INP1, which has been shown to be conserved in several species of key clades among angiosperms [19,20], the degree to which the function of the ELMOD protein family is conserved in other species remains unknown. Further, questions regarding the role of ELMOD_E in the development of round apertures in porate species and/or whether other molecular players are involved in determining the shape of the pollen apertures remain to be determined.

**Figure 1:**
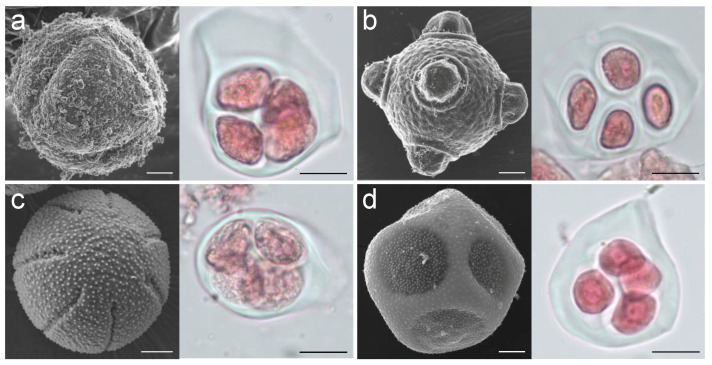
Images showing mature pollen (**left**) and tetrad stage pollen (**right**) for the four species studied. Images of mature pollen were taken with a scanning electron microscope and images of tetrad stage pollen with an optical microscope. Scale bars = 5 μm. (**a**) *Dactylicapnos torulosa*; (**b**) *Fumaria bracteosa*; (**c**) *Eschscholzia californica*; (**d**) *Roemeria refracta*.

In the present study, a comparison of gene expression profiles was made among species with colpate and porate pollen grains during the tetrad stage in order to identify potential genes involved in the determination of aperture shape. The species selected belong to the family Papaveraceae, which is a noteworthy family because of its phylogenetic position at the base of the large clade of the eudicots (order Ranunculales), and is also a euripaline family [21] and therefore allows comparative studies between phylogenetically closely related taxa with different aperture pollen types (reducing the differences in expression due to the phylogenetic relationships). In this study, two pairs of species were used, each pair belonging to a different subfamily of Papaveraceae. Within each pair, one species is colpate and the other porate (Fumarioideae—*Dactylicapnos torulosa* Hook.f. & T. Thomson, 6-colpate, and *Fumaria bracteosa* Pomel, pantoporate; Papaveroideae—*Eschscholzia californica* Cham., 5–7 colpate, and *Roemeria refracta* DC., 6-porate; Figure 1). The de novo transcriptomes assembled and annotated were used to find differentially expressed genes (DEGs) between the colpate and porate species of each pair.

## 2. Results

### 2.1. Transcriptome Sequencing and Assembly

The sequencing of the Ribo-Zero RNA-seq libraries, from anthers with pollen in the tetrad development stage (Figure 1), of the four species yielded 278 million raw paired-end reads (Table S1). Around 98% and 94% of reads presented a phred quality score >20 and >30 (Q20 and Q30), respectively, indicating a high quality of sequences. After filtering out low-quality reads, 133,927,570, 145,092,452, 138,590,018, and 138,351,862 clean reads were obtained for *D. torulosa*, *E. californica*, *F. bracteosa*, and *R. refracta*, respectively (Table S1).

A de novo assembly of reads led to the construction of 294,904, 235,229, and 412,427 contigs with average lengths of 1177, 1321, and 782 bases from *D. torulosa*, *F. bracteosa*, and *R. refracta*, respectively (Table S1). When a single best ORF per transcript longer than 100 amino acids was selected, 127,049, 115,642, and 147,231 transcripts were retained in *D. torulosa*, *F. bracteosa*, and *R. refracta*, respectively. After removing redundant transcripts, 56,689, 52,752, and 92,051 transcripts were kept for further analysis in *D. torulosa*, *F. bracteosa*, and *R. refracta*, respectively (Table S1).

The *E. californica* reads assembly produced 37,400 transcripts after the reads were aligned with the reference genome (Table S1).

### 2.2. Transcriptome Functional Annotation and Classification

**Figure 2:**
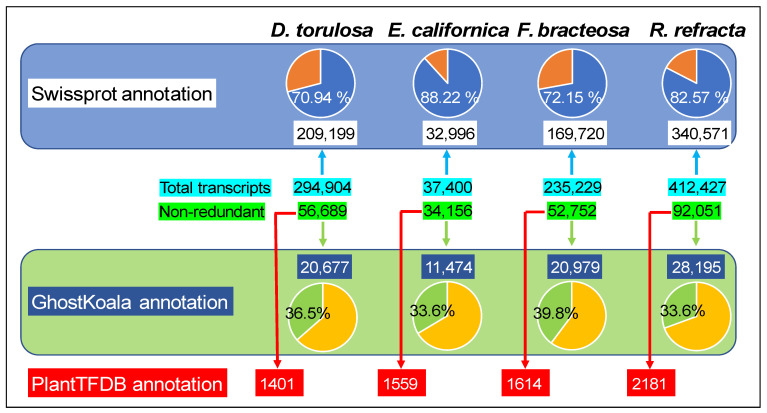
Summary of the number of functionally annotated transcripts for the four species studied, using the Swissprot, GhostKoala, and Plant Transcription Factor databases.

BLAST searches in the SwissProt database with the assembled sequences resulted in 209,199 (70.94%), 32,996 (88.22%), 169,720 (72.15%), and 340,571 (82.57%) annotated transcripts in *D. torulosa*, *E. californica*, *F. bracteosa*, and *R. refracta*, respectively (Figure 2; Tables S1–S5). Using GhostKoala, 20,677 (36.5%; *D. torulosa*), 11,474 (33.6%; *E. californica*), 20,979 (39.8%; *F. bracteosa*), and 28,195 (30.6%; *R. refracta*) entries of non-redundant transcripts were classified in different functional categories, with “Genetic Information Processing” and “Protein Families Involved in Genetic Information Processing” being the most common categories (Figure 2; Table S6). Through PlantTFDB, 1401, 1559, 1614, and 2181 transcripts were identified as transcription factors in *D. torulosa*, *E. californica*, *F. bracteosa*, and *R. refracta*, respectively, with bHLH and B3 being the most abundant families (Figure 2; Table S7).

### 2.3. Differential Gene Expression Analysis

**Figure 3:**
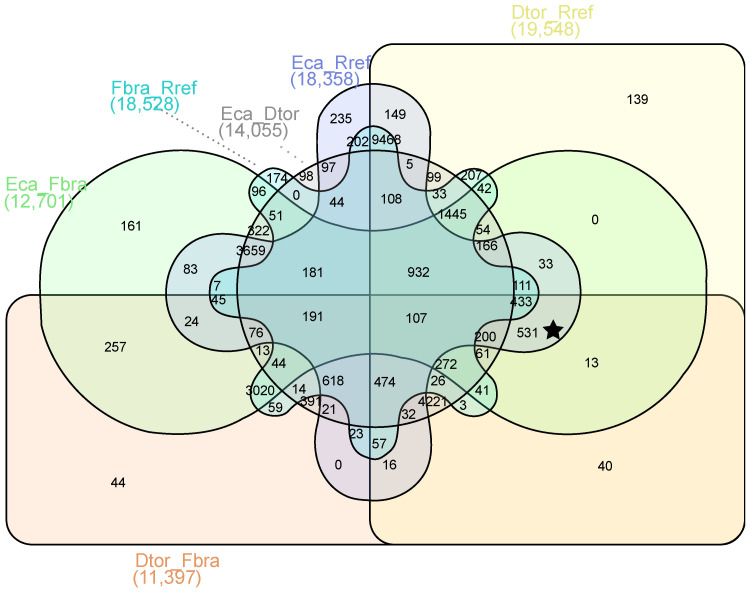
Venn diagram showing the number of DEGs with log_2_FoldChange > 2 and <−2 between the different pairs of species studied. The crossing area indicates the cross-DEGs in different data sets. The star indicates the crossing area, showing the number of DEGs between species with different apertural systems, colpate (*E. californica* and *D. torulosa*) and porate (*R. refracta* and *F. bracteosa*), but not differentially expressed between species with the same apertural system. Dtor, *Dactylicapnos torulosa*; Eca, *Eschscholzia californica*; Fbra, *Fumaria bracteosa*; Rref, *Roemeria refracta*.

In a search for genes potentially involved in the shape of pollen apertures, gene expression profiles were compared at the pollen tetrad stage of colpate vs. porate species. The filtering of the genes identified 531 DEGs among species with different apertural systems (Figure 3). Of these 531 DEGs, 231 were upregulated and 300 were downregulated in colpate species in comparison to porate species (Table S8).

**Figure 4:**
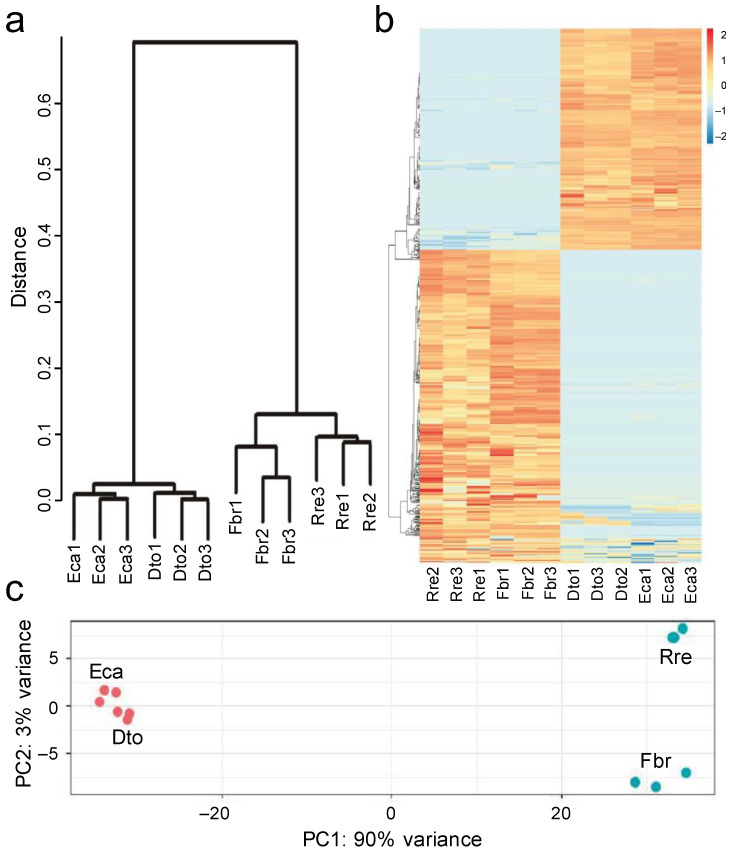
Analysis of differentially expressed genes (DEGs) between colpate pollen and porate pollen species. (**a**) Hierarchical clustering shows dissimilarity among the transcriptome samples; the distance was calculated by Pearson correlation coefficient. (**b**) Heatmap of DEGs between colpate and porate species; the heatmap scale bars indicate log_2_fold changes. (**c**) Principal component analysis of the transcriptome samples. Red points represent colpate species while blue points represent porate species. Eca, *Eschscholzia californica*; Dto, *Dactylicapnos torulosa*; Fbr, *Fumaria bracteosa*; Rre, *Roemeria refracta*.

**Figure 5:**
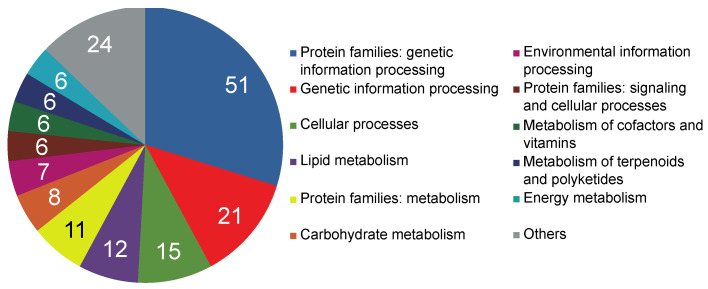
Functional classification of differentially expressed genes (DEGs) between colpate and porate species. Each DEG was classified into KEGG functional categories using GhostKoala mapping tool. Numbers indicate the number of DEGs classified in each category.

**Figure 6:**
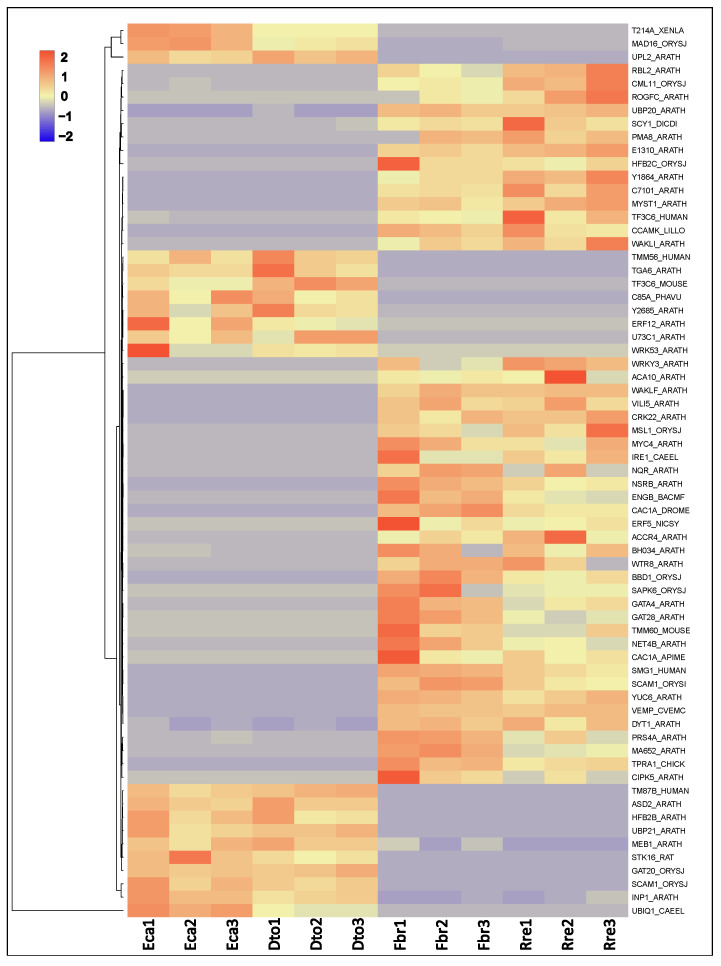
Heatmap of DEGs between colpate and porate species selected as potentially involved in the determination of pollen aperture shape. The heatmap scale bars indicate log_2_fold changes. Eca, *Eschscholzia californica*; Dto, *Dactylicapnos torulosa*; Fbr, *Fumaria bracteosa*; Rre, *Roemeria refracta*.

The hierarchical clustering, principal component analyses, and heatmap revealed DEGs between colpate and porate species samples (Figure 4), irrespective of the Papaveraceae subfamily that they belonged to. The different DEGs were functionally annotated to determine their functions. Through GhostKoala, 171 DEGs (32.2% of the total) were classified in some category, with “Genetic Information Processing” being the most frequent category (Figure 5). Through PlantTFDB, 12 DEGs were identified as transcription factors, repeating the WRKY and ERF transcription factors twice each (Table S9). Using Blast2GO, 420 DEGs were blasted, of which 236 were mapped and 221 were annotated. The 221 annotated DEGs were classified into three categories: biological processes, cellular components, and molecular functions (Table S9). After annotation, 67 genes potentially involved in the determination of pollen aperture shape were selected by manual screening (Table S10; see Section 5 for the screening details). Of these, 46 DEGs were found overexpressed in porate pollen species and 21 in colpate pollen species (Figure 6). It bears highlighting the large number of predicted cellular components identified as integral components of the membrane, while others have been identified as components of the cytoskeleton. Many DEGs are involved in processes of transmembrane transport, regulation of transcription carbohydrate metabolism and organisation, and transport or depolymerisation of components of the cytoskeleton. Important signal transduction elements were categorised, such as presumed serine/threonine protein kinases and transcription factors, and other DEGs displayed DNA-binding and RNA-binding activity. The DEGs included the homologue of *INP1*, which was the first aperture factor discovered, and also genes involved in the deposition or degradation of callose, which are related to the aperture formation process. However, genes of the *ELMOD* family, the only ones described so far which could be involved in the determination of aperture shape, were not found among the DEGs.

### 2.4. ELMOD-like Gene Expression

**Figure 7:**
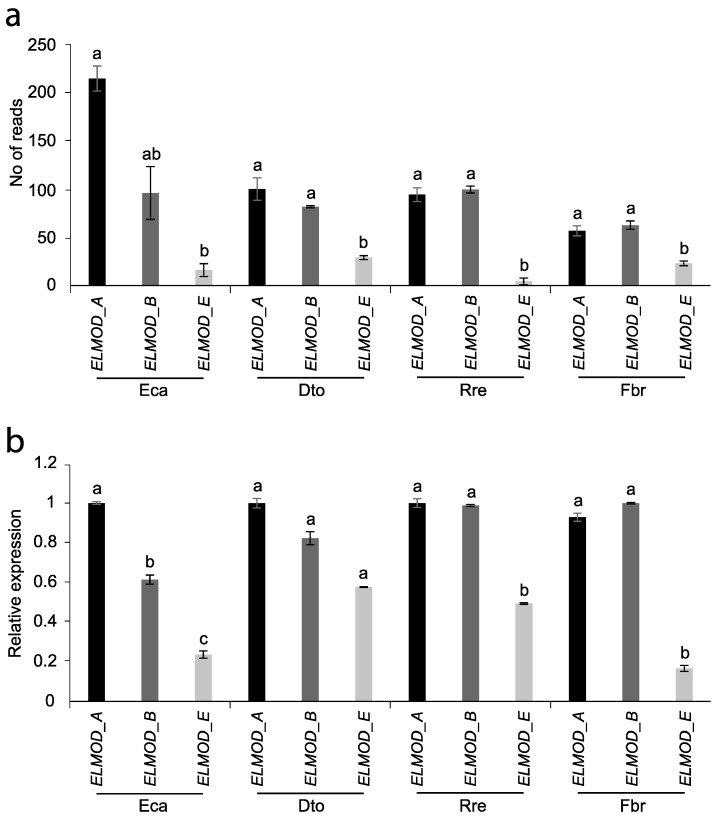
Comparison of *ELMOD*-like gene expression in anthers in the tetrad stage of pollen development. (**a**) *ELMOD*-like gene expression obtained by transcripts quantification. Values correspond to the number of reads per gene quantified using StringTie coverage values. (**b**) *ELMOD*-like gene expression obtained by RT-qPCR analysis. Values on the y-axis correspond to the relative expression intensities, obtained using the 2^ΔΔCt^ method, of each gene in relation to the most highly expressed gene (arbitrary value = 1) within each species. Eca, *Eschscholzia californica*; Dto, *Dactylicapnos torulosa*; Fbr, *Fumaria bracteosa*; Rre, *Roemeria refracta.* Different letters above the bars within each species indicate statistically significant differences in gene expression according to Tukey’s test (*p* < 0.05).

To find out if the different apertural systems (colpate vs. porate) can be determined by the relative expression of the homologue of the *ELMOD_E* gene with respect to the expression of *ELMOD_A*-like and/or *ELMOD_B*-like at the tetrad stage, the expression levels of these three genes were comparatively analysed among the four species studied. Both in the results obtained by RNA-seq analysis and by RT-qPCR, it can be observed that *ELMOD_E*-like has a significantly lower expression than *ELMOD_A*-like and *ELMOD_B*-like for the four species, regardless of the shape of their apertures (Figure 7).

### 2.5. Verification of RNA-Seq Analysis

**Figure 8:**
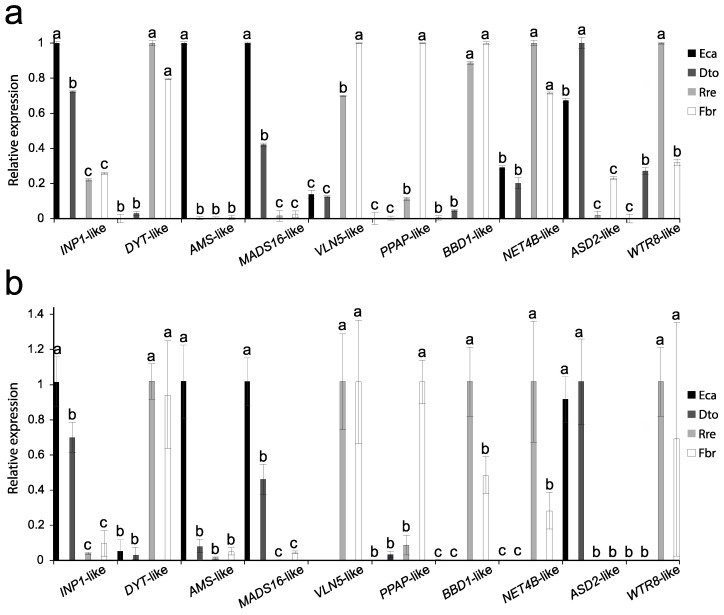
Results of RT-qPCR analysis to confirm the differential expression found by RNA-seq of 10 transcripts among the different species studied. (**a**) Results of RT-qPCR. Values on the y-axis represent the relative expression intensities found using the 2^ΔΔCt^ method. (**b**) Results of RNA-seq. Values on the y-axis represent the relative expression levels, quantified using StringTie coverage values. In (**a**,**b**) for each gene, the expression levels were compared among the homologues of the four species analysed, taking the homologue with maximum expression as reference (arbitrary value = 1). Different letters above the bars within each species indicate statistically significant differences in gene expression according to Tukey’s test (*p* < 0.05). *INP1*-like, *INAPERTURATE POLLEN 1*-like; *DYT*-like, *DYSFUNCTIONAL TAPETUM1*-like; *AMS*-like, *ABORTED MICROSPORE*-like; *MADS16*, *MADS-BOX TRANSCRIPTION FACTOR 16*-like*; VLN5*-like, *VILLIN5*-like; *PPAP*-like, *PUTATIVE PLASMODESMAL ASSOCIATED PROTEIN*-like; *BBD1*-like, *BIFUNCTIONAL NUCLEASE IN BASAL DEFENSE RESPONSE 1*-like; *NET4B*-like, *NETWORKED 4B*-like*; ASD2*-like, *α-L-Arabinofuranosidase*-like*; WTR8*-like, *WAT1-related protein8*-like. Eca, *Eschscholzia californica*; Dto, *Dactylicapnos torulosa*; Fbr, *Fumaria bracteosa*; Rre, *Roemeria refracta*.

For verification of the results found by RNA-seq, a RT-qPCR was used to confirm the differential expression of 10 DEGs, which potentially were involved in genetic determination of the apertural system. The RT-qPCR results were consistent with those of the RNA-seq analysis (Figure 8). The homologues of *DYSFUNCTIONAL TAPETUM1* (*DYT1*), *VILLIN5* (*VLN5*), *PUTATIVE PLASMODESMAL ASSOCIATED PROTEIN* (*PPAP*), *BIFUNCTIONAL NUCLEASE IN BASAL DEFENSE RESPONSE 1* (*BBD1*), *NETWORKED 4B* (*NET4B*), and *WAT1-related protein8* (*WTR8*) were more expressed in porate pollen (*R. refracta* and *F. bracteosa*). Meanwhile, the homologues of *MADS-BOX TRANSCRIPTION FACTOR 16* (*MAD16*), *INAPERTURATE POLLEN 1* (*INP1*), and *α-L-Arabinofuranosidase* (*ASD2*) were more expressed in colpate pollen (*D. torulosa* and *E. californica*). Finally, the homologue of the transcription factor *ABORTED MICROSPORES* (*AMS*) was more expressed in *E. californica* than in other species (Figure 8). These findings support the results found by RNA-seq analysis.

## 3. Discussion

Pollen apertures play an important role in the reproductive process of spermatophytes, favouring the viability and germination of the male gametophyte. Evolutionary changes in the aperture system have come to be interpreted as key innovations that have led to the success of certain groups such as the Eudicots [1]. The shape of the aperture is often related to the tolerance to dehydration of the male gametophyte. Furrow-like apertures tend to close and isolate the gametophyte from the dry external environment, even gradually with varying degrees of dryness, whereas pore-like apertures cannot adequately seal the aperture [9,10,11]. However, the distribution of porate pollen throughout most of the angiosperm groups, including species from xeric environments, suggests some kind of selection of this apertural type, which could be related to a higher germination speed of the porate pollen because it does not completely dehydrate before dispersion, and which could lead to a higher reproductive success in the short term [12,13]. Whether this process involves the selection of a molecular mechanism common to angiosperms for the emergence of pore phenotypes is unknown. The molecular basis of pollen aperture shape remains to be elucidated. In this study, we used the natural variation of apertural morphology in Papaveraceae to adopt a comparative perspective, between colpate and porate species, to find candidate molecular players involved in apertural shape determination and their conservation among taxonomic groups.

Recently, Zhou et al. [18] discovered some members of the ELMOD protein family involved in the control of early steps in aperture domain formation in *Arabidopsis*. While the ELMOD_A and ELMOD_B/MCR paralogues specify position and number of aperture domains with redundancy in function, ELMOD_E can interact with MCR and ELMOD_A activities, changing the aperture morphology from colpus to pore, although this does not naturally occur in *Arabidopsis* [18]. *ELMOD_A* and *MCR* are both expressed at or near the tetrad stage of pollen development. Experiments by the same authors showed that the neomorphic activity of ELMOD_E occurs only when gene expression levels are elevated during the tetrad phase; however, during this stage, *ELMOD_E* expression is low and therefore does not influence the colpate apertures of *Arabidopsis*. Thus, differential expression of these *ELMOD* genes could be a way to regulate the occurrence of porate pollen. *ELMOD_A* and *MCR* are paralogues that diverged in the common ancestor of the Brassicaceae [18]; however, many species from the main angiosperm groups included in the “A/B clade” of the phylogeny in Zhou et al. [18] have at least two paralogues of this lineage. These authors suggested that these independent duplications could reflect a strong selective pressure to maintain more than one A/B gene and their redundancy. *ELMOD_E* corresponds to a basal ELMOD lineage. Homologues of all three *ELMOD* genes (2 A/B genes and *ELMOD_E*-like) were found in the four transcriptomes of the present work, but they were not differentially expressed between morphs at the tetrad stage investigated. Furthermore, as in *Arabidopsis*, in the species analysed in this study, the *ELMOD_E*-like gene is less expressed than *ELMOD_A*-like or *ELMOD_B*-like/*MCR*-like regardless of the shape of their apertures. The key aspect of this result is that it does not support the hypothesis that the interaction of ELMOD_E with the proteins of the A/B lineage regulates the change from colpus to pore in Papaveraceae (a basal eudicot), and thus the conserved function of ELMOD_E. The coexpression of A/B lineage genes during the tetrad stage agrees with what has been observed in *Arabidopsis* and their possible redundant role in determining aperture domains, although further studies are needed to ascertain whether their function is conserved in other plant groups.

Although ELMOD_E can determine aperture morphology in *Arabidopsis* [18], the results of the present study suggest that this is not the case in basal eudicots. The transcriptome analysis here comparing species with colpate and porate pollen presents a basis for identifying DEGs that may represent candidates involved in aperture morphology in the tetrad stage.

Of the 531 DEGs among species with different apertural systems, 56.5% were upregulated in the porate species. The proportion of overexpressed genes in the porate species was even higher (68.66%) when we consider the 67 DEGs involved in processes of pollen aperture and pollen wall development, suggesting a greater activation of genes potentially involved in the determination of pollen aperture shape in the porate species. Among these 67 DEGs, there are genes involved in callose patterning, cytoskeleton organisation-related genes, receptor like-kinase, or serine/threonine-protein kinase, mostly overexpressed in porate species; genes involved in wall remodelling, overexpressed in colpate species; and a similar number of genes encoding integral membrane proteins, including transporters and transcription factors, overexpressed in both colpate and porate species.

One of the DEGs, *INP1*, was found to be involved in aperture system determination and has been seen to affect aperture morphology to some extent, but only when its expression levels are low. In these cases, furrow-like apertures are significantly reduced in length, a finding reported elsewhere in *Arabidopsis* and *Eschscholzia californica* [14,20]. The homologue of *INP1* is one of the DEGs in the present work to be identified among colpate and porate species, being downregulated in the porate species. The direct involvement of *INP1* in the change from colpus to pore is unlikely, as different complementation experiments performed by transforming *Arabidopsis* with *INP1* from other species with different apertures resulted in the typical *Arabidopsis* apertures [17,19]. It is possible that the differential expression of *INP1*-like is related to the smaller surface area of the pore-like apertures. In the tetrad stage, INP1 accumulates in the plasma membrane of pollen at the sites where the apertures later appear. Then, INP1 prevents the deposition of sporopollenin in these areas, and the apertures are formed. Thus, the porate pollen needs less protein to cover the pre-established aperture domains than in the case of colpate pollen.

However, the homologue of the recently described *INAPERTURATE POLLEN 2* (*INP2*), a species-specific partner for *INP1* in *Arabidopsis*, is upregulated in porate species, but is also differentially expressed between the two porate species. Therefore, it does not show a similar expression pattern to *INP1* as would be expected because these two proteins interact with each other for aperture formation and have coevolved [17]. Thus, perhaps the function of *INP2* is not conserved in angiosperms as a whole, or perhaps in these species, another species-specific partner of INP1 fulfils the function of INP2.

The callose wall appears to play an essential role in the development of apertures [22]. Apertures develop where membrane ridges maintain close contact with the callose wall, avoiding primexine deposition and thus preventing exine development; INP1 appears to be involved in maintaining these ridges near the callose wall [22]. In addition, it has been shown that the position of the apertures is related to where additional depositions of callose are formed after cytokinesis, and that these depositions have the same shape as the future apertures, suggesting that they must determine the shape of the aperture [23,24]. So far, no molecular factor involved in these deposits has been found. It bears mentioning that the present study identified five DEGs annotated as homologues of genes that in some way affect the deposition or degradation of callose. One of these, upregulated in porate pollen, is a homologue of *DYSFUNCTIONAL TAPETUM1* (*DYT1*), which encodes a bHLH transcription factor. *DYT1* is strongly expressed in the tapetum, where it is important for the expression of about 1000 anther genes, including genes involved in callose synthesis and degradation, and pollen wall development [25,26]. *DYT1* expression declines rapidly at the end of meiosis of meiocytes. The *dyt1* mutant exhibits abnormal anther morphology, and meiocytes do not have a thick callose wall although they are able to complete meiosis [27]. According to Feng et al. [25], DYT1 possibly regulates genes that are essential for active metabolism in the tapetal cells and for the export of materials to meiocytes, such as those for callose wall formation. More research is needed to establish whether it affects the additional deposition of callose that determines the position and perhaps the shape of the apertures. Notably, in the study by Feng et al. [25] on the regulation exerted by DYT1 on *Arabidopsis* anther genes, one of the genes downregulated in *dyt1* knockout mutants, and mutants of its paralogue *AMS* (*ABORTED MICROSPORES*), was *ELMOD_E* (see Tables S1 and S11 in [25]). This directly links *DYT1* and *AMS* to the only gene known to affect aperture morphology in *Arabidopsis*. It has been conjectured that tapetum transcription factors may affect the development of the pollen wall. Lou et al. [28] showed a direct regulation by *AMS* of tapetum genes for the sexine and nexine formation, indicating that a transcriptional cascade in the tapetum specifies the development of the pollen wall. Thus, *ELMOD_E* could be one of the targets of this transcriptional cascade. One of the genes which is regulated by *AMS* and specifies the formation of the nexine layer, *TRANSPOSABLE ELEMENT SILENCING* VIA *AT-HOOK* (*TEK*), has recently been shown to be directly involved in callose synthesis by negatively regulating *CALLOSE SYNTHASE5* (*CalS5*) after the tetrad stage [29], when the callose wall is believed to begin to be degraded by tapetally secreted callase activity [30].

Among the DEGs of the present study, two β-1,3-glucanases were found to be upregulated in porate pollen, which are involved in callose degradation. One of these, annotated as Glucan endo-1,3-beta-glucosidase 10/AtBG-pap and encoded by the gene *PUTATIVE PLASMODESMAL ASSOCIATED PROTEIN* (*PPAP*) (At5g42100) [31], is a plasmodesmata-associated membrane protein involved in plasmodesmal callose degradation. This β-1,3-glucanase could be recruited, in addition to plasmodesmata, to other specific membrane domains such as additional callose deposits, where it would act by degrading callose in porate pollen and thus help determine the shape of the apertures. However, this needs further study to be confirmed. The other β-1,3-glucanase was annotated as *β-1*,*3-GLUCANASE 5*/*BG5*, whose protein is located outside the cell [32]. 

Two genes involved in the callose deposition were annotated as the two homologues of *Oryza sativa BIFUNCTIONAL NUCLEASE IN BASAL DEFENSE RESPONSE 1* (*OsjBBD1*) and *BIFUNCTIONAL NUCLEASE IN BASAL DEFENSE RESPONSE 2* (*OsjBBD2*), nucleases with both RNase and DNase activities. BBD1 functions in cell wall reinforcement through the abscisic acid-derived callose deposition that is induced following infection by a necrotrophic pathogen, probably through activation of *PDF1.2*, *ABA1*, and *AtSAC1* gene expression [33]; the function of BBD2 is not clear but appears to be redundant to BBD1 [34]. The results of the present study indicate that *BBD1*-like was upregulated in porate pollen, while *BBD2*-like was upregulated in colpate pollen, suggesting differential dominance according to the type of apertures. The role of this gene in pollen is unknown.

Thus, any of these genes that directly or indirectly affect the deposition and degradation of callose could act by determining the shape of additional callose deposition and thereby affect the shape of the apertures.

Among the Go categories, it was found in the present study that many DEGs are integral components of the membrane. Seven of these, i.e., two upregulated in colpate pollen and five upregulated in porate pollen, code for transmembrane transporters. Two of the five upregulated in porate pollen were annotated as belonging to the plant drug/metabolite exporter (DME) family, homologues of the *Arabidopsis* genes *WAT1(WALLS ARE THIN1)-related protein7* (*WTR7*) and *WAT1-related protein8* (*WTR8*). WAT1 is required for secondary cell wall deposition [35], and this and other proteins of this family are auxin-induced proteins involved in different developmental processes [35,36,37]. In *Arabidopsis*, this protein family involves at least 38 members, most of unknown function; according to Busov et al. [36], this multigene structure suggests functional divergence of its members. Because the callose wall (surrounding the microspore mother cell (MMC) and tetrads) is quite impermeable to many primexin components and other components involved in exine patterning [22], most exine precursors must be synthesised by the MMCs and/or microspores and transported across the membrane to occupy their final positions. Thus, differentially expressed transmembrane transporters suggest the need to transport different factors, or in different quantities, depending on the aperture morphology. 

DEGs were found to be involved in the organisation of actin filaments, the cytoskeleton, and microtubules, while others had an actin-binding function, and still others served as components of microtubules or the cytoskeleton. Several studies have described a relationship between the organisation of cytoskeletal elements and the formation of apertures. In *Nicotiana*, the distribution of microtubules in postmeiotic cytokinesis is related to the number of apertures [38]. *Vigna* shows a spatial correlation between microtubules and exine, with cytoplasmic patches of microtubules appearing where apertures form during the tetrad stage [39]. In *Lilium henryi*, the microtubular organising centres (MTOCs) participate in locating the endoplasmic reticulum in the area where the aperture later appears, acting as a barrier for the deposition of sporopollenin [40]. In addition, a certain organisation of the microtubules can cause undulations in the plasma membrane, which is associated with the construction process of the primexine framework and the apertures [41]. 

One of the DEGs of the present work, related to the cytoskeleton and upregulated in porate pollen, was annotated as a homologue of *NETWORKED 4B* (*NET4B*) of *Arabidopsis*, a member of the Networked (NET) superfamily. NET proteins possess an actin-binding region (NAB domain) and are membrane-associated. These proteins specifically link actin filaments to cell membranes to specify different membrane domains [42]. In *Arabidopsis*, the NET superfamily is composed of four subfamilies (NET1-4). *NET1A* is a gene highly expressed in plasmodesmata, where the actin cytoskeleton, plasma membrane, and endoplasmic reticulum are brought together. Plasmodesmata are small and defined areas in walls, such as apertures. Genes from the *NET2* subclade are expressed preferentially in pollen, which could indicate interactions with pollen-specific ligands [43]. NET4A protein localises to highly constricted regions of the vacuolar membrane and contributes to vacuolar morphology [43]. 

Another DEG involved in cytoskeleton organisation was annotated as a homologue of *VILLIN5* (*VLN5*), which is preferentially expressed in *Arabidopsis* pollen, and which is upregulated in porate pollen. VLN5 is an actin-binding protein that plays a key role in the formation of higher-order structures from actin filaments and in the regulation of actin dynamics in eukaryotic cells. Villin family members from plants have been shown to sever, cap, and bundle actin filaments [44]. The loss of VLN5 function retards pollen tube growth, and actin filaments are more sensitive to depolymerisation in *Arabidopsis* pollen grains and tubes [44]. In addition, VLN5 functions in concert with oscillatory calcium gradients in pollen. In relation to this, in the present work, different DEGs were found to be related to calcium, such as calcium transporters, calcium-binding proteins, or calcium channels.

## 4. Conclusions

Comparative transcriptome analysis of two pairs of Papaveraceae species belonging to two different subfamilies and with different apertural systems within each pair (Fumarioideae—*Dactylicapnos torulosa*, 6-colpate, and *Fumaria bracteosa*, pantoporate; Papaveroideae—*Eschsholzia californica*, 5–7 colpate, and *Roemeria refracta*, pantoporate) enabled the identification of genes potentially involved in the determination of aperture shape. In total, 531 DEGs were found among species with different apertural systems, including genes involved in processes key to aperture formation such as the synthesis or degradation of callose, or the organisation of cytoskeletal elements. The Papaveraceae homologue of the *ELMOD_E* gene, the only one described so far that could be involved in determining aperture shape in *Arabidopsis*, is not differentially expressed between colpate and porate species during the tetrad stage. Furthermore, in none of the species analysed does *ELMOD_E*-like show higher expression than the other two members of the A/B lineage gene family, *ELMOD_A*-like and *ELMOD_B*-like. All this evidence fails to uphold the hypothesis, supported in *Arabidopsis*, stating that the interaction between ELMOD_E and A/B lineage proteins regulates colpo-to-pore shift in Papaveraceae, and thus the conserved function of ELMOD_E. Finally, the transcriptomes of anthers with pollen in the tetrad developmental stage of *D. torulosa*, *R. refracta*, and *F. bracteosa* species were obtained for the first time in this study. These data will be useful for future studies in the field of floral evolution and development.

## 5. Materials and Methods

### 5.1. Plant Material

Plants of *Dactylicapnos torulosa*, *Eschscholzia californica*, *Fumaria bracteosa*, and *Roemeria refracta* were sown in pots (9 × 9 × 9 cm) with universal substrate and vermiculite mixed in a 3:1 ratio and kept in a greenhouse at a temperature range between 26 °C and 14 °C under a light/dark cycle of 16/8 h. Each pot was fertilised once at the beginning of the experiment and watered daily. To confirm the pollen development stage for each species, pollen contained in buds of different sizes was stained with basic fuchsin and observed by optical microscopy Olympus-CX31 (Olympus Corporation, Tokyo, Japan). When pollen was at the tetrad stage (Figure 1), buds (≈1 mm in diameter, *D. torulosa*; ≈4 mm in diameter, *E. californica*; ≈0.4 mm, *F. bracteosa*; and ≈3 mm, *R. refracta*) were collected from three plants per species (three independent biological replicates), and anthers were removed with a cooled scalpel, frozen in liquid nitrogen, and stored at −80 °C until use.

To document the apertural system of the four species studied, we obtained pollen images by scanning electron microscopy (SEM). For this purpose, anthers of each species were fixed according to Fernández et al. [45], and pollen was observed with a scanning electron microscope (model SMT; Zeiss, Jena, Germany) at the Centro de Instrumentación Científica (University of Granada).

### 5.2. RNA Extraction, Library Construction, Sequencing, and Read Filtering

Total RNA was extracted from 500 mg of anthers with pollen in the tetrad developmental stage, using the NucleoSpin^®^ RNA Plant kit (Macherey-Nagel GmbH and Co., Ltd., Düren, Germany), following the manufacturer’s instructions. Quantity and quality were ensured using a Nanovue Plus Spectrometer (Biochrom, Cambridge, UK) and by agarose gel electrophoresis. Library preparation and sequencing were performed at Macrogen Inc. (Seoul, Republic of Korea). RNA libraries were prepared with an Illumina TruSeq Stranded Total RNA sample Preparation kit with Ribo-Zero Plant and sequenced on an Illumina Hiseq 2500 platform with paired-end reads of 150 bases. The raw data were generated using Illumina package bcl2fastq.

The quality control of raw single reads (in FASTQ format) was evaluated using FastQC v0.11.83 (http://www.bioinformatics.bbsrc.ac.uk/projects/fastqc (accessed on 20 February 2020)). TrimGalore v0.6.4 (https://github.com/FelixKrueger/TrimGalore (accessed on 25 February 2020); parameters: --paired --phred33 -e 0.1 -q 20) was used for removing adaptors and low-quality sequences from the data set. Unpaired reads were also discarded for the remainder of the assembly pipeline. After trimming, FastQC was used again to examine the characteristics of the libraries and to verify trimming efficiency.

### 5.3. De Novo Transcriptome Assembly and Transcript Reconstruction

The high-quality reads from *D. torulosa*, *F. bracteosa*, and *R. refracta* were de novo assembled by Trinity v1.8 ([46]; parameters: --seqType fq --JM 10G\--left reads.ALL.left.fq –right reads.ALL.right.fq\--SS_lib_type RF --CPU 6). In the case of *E. californica*, given that its genome is available (ftp://ftp.kazusa.or.jp/pub/Eschscholzia/ECA_r1.0.cds.fa.gz (accessed on 2 March 2020)), it was used as reference.

All reads across three biological replicates from each species were combined to generate a single reference Trinity assembly per species. Basic statistical information over de novo assemblies was gained by running the Trinity package utility script TrinityStats.pl.

Then, a transcript reconstruction was carried out to filter the best-generated transcripts for de novo assembled species. For this task, TransDecoder v5.5.0 ([47]; https://transdecoder.github.io/ (accessed on 15 March 2020)) was used to select the single best open-reading frame (ORF) per transcript longer than 100 amino acids through the command TransDecoder.LongOrfs, and to predict the coding sequences with the command TransDecoder.Predict.

As a means of compiling non-redundant transcripts, highly similar coding sequences were clustered using CD-HIT v4.8.1 [48] with an amino acid sequence identity threshold of 0.99.

### 5.4. Transcript Quantification

RNA-seq reads of different samples were aligned with their relevant Trinity assembly references, or genome reference in the case of *E. californica*, using the software HISAT2 v2.1.0 (https://ccb.jhu.edu/software/hisat2/ (accessed on 19 March 2020); [25]). The aligned reads were assembled and quantified using the software StringTie v2.0 (https://ccb.jhu.edu/software/stringtie/ (accessed on 21 March 2020)), with the option for merging the assemblies of three biological samples [25]. Assembly information was obtained through the GffCompare v0.10.1 program (https://ccb.jhu.edu/software/stringtie/gffcompare.shtml (accessed on 29 March 2020); [49]).

### 5.5. Transcriptome Annotation and Functional Classification

The transcripts were annotated using the Trinotate annotation pipeline following the method outlined at http://trinotate.github.io/ (accessed on 14 April 2020). Initially, they were searched against the SwissProt database ([50]; https://data.broadinstitute.org/Trinity/__deprecated_trinotate_resources/Trinotate_v3_RESOURCES/uniprot_sprot.pep.gz (accessed on 18 April 2020)) using BLASTX, allowing one hit and with output in tabular format. The expected protein translations were attained using TransDecoder and then searched against SwissProt using BLASTP. The same BLAST parameters were used as for the BLASTX searches. The BLAST searches were loaded into the Trinotate SQLite database v3.0.2 (http://trinotate.github.io/ (accessed on 23 April 2020)), and an annotation report was generated. An e-value of 1 × 10^−5^ was used as the threshold for the BLAST results during the report generation.

In an effort to assign a function with each transcript, annotated transcripts were further functionally classified with the Gene Ontology (GO; [51]) and Kyoto Encyclopedia of Genes and Genomes (KEGG; [52]) databases using the Blast2GO v5.2.5 [53] and GhostKoala mapping tools [54], respectively. For the prediction of putative plant transcription factor (TF) among transcripts, coding sequences were aligned to TF domains from Plant Transcription Factor Database (PlantTFDB—Plant Transcription Factor Database @ CBI, PKU; [55]).

### 5.6. Analysis of DEGs

The gene expression profiles of two colpate pollen species (*D. torulosa* and *E. californica*) were compared with two porate pollen species (*F. bracteosa* and *R. refracta*). Differential gene expression analyses were performed with the DESeq2 R package (v1.24.0), using the coverage produced by StringTie and the merging transcript of different species by annotation made with the SwissProt database using BLASTX. DEGs were filtered considering the *p*-value and *p*-adj < 0.05 and the log_2_-fold change >2, <−2. DEGs between species with different apertural systems, colpate (*E. californica* and *D. torulosa*) and porate (*R. refracta* and *F. bracteosa*), but not differentially expressed between species of the same apertural system were selected. The Venn diagram was generated to show the mutual and nonmutual DEGs among the different pairs of species using the web-based tool InteractiVenn [56]. Selected DEGs were classified functionally using the GhostKoala mapping tool, as described above. For the hierarchical clustering of log-transformed expression data, principal component analysis, and the heatmap, the DESeq2 R package (v1.24.0) was used.

To screen for DEGs potentially involved in determining pollen aperture shape, a manual search for pollen-specific genes and/or genes involved in processes of pollen aperture and pollen wall development was performed based on information provided by the functional annotation, UniProt website, and gene literature.

### 5.7. Expression Analysis of ELMOD Genes

To determine if differences in *ELMOD_E* expression or its expression relative to *ELMOD_A* and/or *ELMOD_B* at the tetrad stage can determine the shape of apertures, the expression levels of these three genes were comparatively analysed among the four species studied. For the identification of the ELMOD sequences of the four species of interest, they were initially searched against the *E. californica* genome database (ftp://ftp.kazusa.or.jp/pub/Eschscholzia/ECA_r1.0.cds.fa.gz (accessed on 23 May 2020)) using the *A. thaliana* protein sequences and BLASTP. Then, the sequences of *E. californica* were used to perform BLASTX and BLASTN searches on the Trinity assembly reference created for each of the other species. The expression of each gene for each species was then quantified by both StringTie and also by RT-qPCR, following the methodology described in the next section. Data were analysed by employing one-way analysis of variance with repeated measures using Tukey’s pairwise comparison test with the XRealStats add-in for Excel.

### 5.8. Verification of RNA-Seq Analysis by RT-qPCR

The differences observed in gene expression were verified by RT-qPCR performed for 10 selected DEGs. RNA of anthers with pollen in the tetrad developmental stage was extracted as described above, and 1 μg of RNA was reverse-transcribed to cDNA using SuperScript III Reverse Transcriptase (Invitrogen, Karlsruhe, Germany) and oligo(dT)18 (ThermoFisher Scientific, Waltham, MA, USA). The cDNA was diluted to 50 ng/μL and used to perform the RT-qPCR. *ACTIN* served as the reference gene for calculating the relative expression intensities in RT-qPCR analyses, using the 2^ΔΔCt^ method [57]. The RT-qPCR was carried out using the FastGene IC Green 2× qPCR mix (NIPPON Genetics, Tokyo, Japan), according to the manufacturer’s instructions, and the qTower 2.2 real-time PCR thermocycler (Analytik, Jena, Germany). Gene-specific primers were designed using the software Primer3 ([58]; Table S11). All experiments were repeated with three biological and three technical replicates. Data were analysed with a one-way analysis of variance with repeated measures using Tukey’s pairwise comparison test.

## Data Availability

All raw sequences for transcriptomes are available in the GenBank database under the accession numbers SRR19536723–SRR19536725 for *Dactylicapnos torulosa* samples, SRR19536720–SRR19536722 for *Fumaria bracteosa* samples, and SRR19536717–SRR19536719 for *Roemeria refracta* samples and in the European Nucleotide Archive (ENA) database under the accession numbers ERS6376182–ERS6376184 for *Eschscholzia californica* samples.

## Supplementary Materials

The following supporting information can be downloaded at https://www.mdpi.com/article/10.3390/plants12071570/s1, Supplementary Tables S2–S5, S7–S9 are available at Digibug repository http://hdl.handle.net/10481/71277 (accessed on 1 January 2023). Table S1: Summary of sequencing, assembly and annotation of *Dactylicapnos torulosa*, *Fumaria bracteosa*, *Roemeria refracta* and *Eschscholzia californica*. Table S2: Trinotate annotation report for *Dactylicapnos torulosa*. Annotation through blastx and blastp for predict transcript against Swissprot database. Table S3: Trinotate annotation report for *Fumaria bracteosa*. Annotation through blastx and blastp for predict transcript against Swissprot database. Table S4: Trinotate annotation report for *Roemeria refracta*. Annotation through blastx and blastp for predict transcript against Swissprot database. Table S5: *Eschscholzia californica* annotation through blastx against Swissprot database for transcripome transcript aligned to reference. Qseqid, query or source (e.g., gene) sequence id; seqid, subject or target (e.g., reference genome) sequence id; pident, percentage of identical matches; length, alignment length (sequence overlap); mismatch, number of mismatches; gapopen, number of gap openings; qstart, start of alignment in query; qend, end of alignment in query; sstart, start of alignment in subject; send, end of alignment in subject; evalue, expect value; bitscore, bit score. Table S6: Functional classification for transcripts of *Fumaria bracteosa*, *Roemeria refracta*, *Dactylicapnos torulosa* and *Eschscholzia californica* transcriptome assembly. Classification into KEGG functional categories, using GhostKoala mapping tool. Table S7: Transcripts annotated as transcription factor through PlantTFDB. Table S8: Genes differentially expressed between colpate and porate species. Annotation through BLASTX searching against the SwissProt Database. Table S9: Functional classification of differentially expressed genes (DEGs) between colpate and porate species using Blast2GO software. Sheet 1, Blast2GO output file with annotations and functional classification for each DEG. Sheet2, summary of the number of DEGs for each functional annotation within the three different functional categories, note that each DEG can have several annotation possibilities. Table S10: DEGs among porate and colpate pollen species filtered for their characteristics as potential players in determining pollen aperture morphology. Swiss_Prot Code, assigned entry by Blast again SwissProt database; TF, transcription factors identified by PlantTFDB. Table S11: Primer sequences used in this study.
