# Supplementary material for: Identification of Candidate Genes Involved in the Determinism of Pollen Grain Aperture Morphology by Comparative Transcriptome Analysis in Papaveraceae
Source: Plants (Basel). 2023 Apr 6;12(7):1570. doi: 10.3390/plants12071570 (PMC10096813; doi:10.3390/plants12071570)
Supplement: Supplementary file 1 [file plants-12-01570-s001.zip › plants-2181552-supplementary.docx]

**Supporting Information**

**Plants**

**Identification of candidate genes involved in the determinism of pollen grain aperture morphology by comparative transcriptome analysis in Papaveraceae**

**Ismael Mazuecos-Aguilera, Víctor N. Suárez-Santiago^*^**

Department of Botany, Faculty of Sciences, University of Granada, 18071 Granada, Spain

* Correspondence: vsuarez@ugr.es

**Table S1.** Summary of sequencing, assembly and annotation of *Dactylicapnos torulosa, Fumaria bracteosa, Roemeria refracta* and *Eschscholzia californica.*

**Table S2.** Trinotate annotation report for *Dactylicapnos torulosa*. Annotation through blastx and blastp for predict transcript against Swissprot database.

Available at Digibug repository <http://hdl.handle.net/10481/71277>

**Table S3.** Trinotate annotation report for *Fumaria bracteosa*. Annotation through blastx and blastp for predict transcript against Swissprot database.

Available at Digibug repository <http://hdl.handle.net/10481/71277>

**Table S4.** Trinotate annotation report for *Roemeria refracta*. Annotation through blastx and blastp for predict transcript against Swissprot database.

Available at Digibug repository <http://hdl.handle.net/10481/71277>

**Table S5.** *Eschscholzia californica* annotation through blastx against Swissprot database for transcripome transcript aligned to reference. Qseqid, query or source (e.g., gene) sequence id; seqid, subject or target (e.g., reference genome) sequence id; pident, percentage of identical matches; length, alignment length (sequence overlap); mismatch, number of mismatches; gapopen, number of gap openings; qstart, start of alignment in query; qend, end of alignment in query; sstart, start of alignment in subject; send, end of alignment in subject; evalue, expect value; bitscore, bit score.

Available at Digibug repository <http://hdl.handle.net/10481/71277>

**Table S6.** Functional classification for transcripts of *Fumaria bracteosa, Roemeria refracta, Dactylicapnos torulosa* and *Eschscholzia californica* transcriptome assembly. Classification into KEGG functional categories, using GhostKoala mapping tool.

| Specie  Category | *Fumaria bracteosa* | *Roemeria refracta* | *Dactylicapnos torulosa* | *Eschscholzia californica* |
| --- | --- | --- | --- | --- |
| Protein families: genetic information processing | 4780 | 6276 | 4751 | 2264 |
| Genetic Information Processing | 3668 | 6425 | 3758 | 2270 |
| Carbohydrate metabolism | 1489 | 1706 | 1528 | 948 |
| Protein families: signaling and cellular processes | 1467 | 1727 | 1451 | 682 |
| Protein families: metabolism | 1466 | 1736 | 1510 | 682 |
| Environmental Information Processing | 1213 | 1948 | 1146 | 754 |
| Cellular Processes | 965 | 1062 | 940 | 518 |
| Lipid metabolism | 768 | 878 | 689 | 429 |
| Unclassified: metabolism | 753 | 866 | 779 | 425 |
| Organismal Systems | 706 | 852 | 644 | 451 |
| Amino acid metabolism | 640 | 957 | 663 | 399 |
| Metabolism of cofactors and vitamins | 522 | 593 | 467 | 229 |
| Energy metabolism | 427 | 439 | 383 | 334 |
| Glycan biosynthesis and metabolism | 426 | 360 | 390 | 162 |
| Human diseases | 408 | 784 | 434 | 250 |
| Metabolism of terpenoids and polyketides | 355 | 320 | 312 | 139 |
| Nucleotide metabolism | 273 | 339 | 286 | 158 |
| Byosinthesis of other secondary metabolites | 243 | 390 | 188 | 140 |
| Unclassified | 144 | 167 | 96 | 46 |
| Unclassified: signaling and cellular processes | 134 | 137 | 123 | 93 |
| Metabolism of other aminoacids | 85 | 188 | 84 | 70 |
| Unclassified: genetic information processing | 35 | 38 | 50 | 25 |
| Xenobiotics degradation and metabolism | 9 | 8 | 5 | 6 |
| Total | 20,979 (39.8%) | 28,195 (30.6%) | 20,677  (36.5%) | 11,474 (33.6%) |
| Entries | 52,752 | 92,051 | 56,689 | 34,156 |

**Table S7.** Transcripts annotated as transcription factor through PlantTFDB.

Available at Digibug repository <http://hdl.handle.net/10481/71277>

**Table S8.** Genes differentially expressed between colpate and porate species. Annotation through BLASTX searching against the SwissProt Database.

Available at Digibug repository <http://hdl.handle.net/10481/71277>

**Table S9.** Functional classification of differentially expressed genes (DEGs) between colpate and porate species using Blast2GO software. Sheet 1, Blast2GO output file with annotations and functional classification for each DEG. Sheet2, summary of the number of DEGs for each functional annotation within the three different functional categories, note that each DEG can have several annotation possibilities.

Available at Digibug repository <http://hdl.handle.net/10481/71277>

**Table S10.** DEGs among porate and colpate pollen species filtered for their characteristics as potential players in determining pollen aperture morphology. Swiss_Prot Code, assigned entry by Blast again SwissProt database; TF, transcription factors identified by PlantTFDB

| **Swiss_Prot Code** | **Name** | **TF** |
| --- | --- | --- |
| DYT1_ARATH | DYT1_ARATH Transcription factor DYT1 {ECO:0000305} |  |
| E1310_ARATH | E1310_ARATH Glucan endo-1,3-beta-glucosidase 10 |  |
| BBD1_ORYSJ | BBD1_ORYSJ Bifunctional nuclease 1 |  |
| ASD2_ARATH | ASD2_ARATH Alpha-L-arabinofuranosidase 2 |  |
| WTR8_ARATH | WTR8_ARATH WAT1-related protein At1g44800 |  |
| NET4B_ARATH | NET4B_ARATH Protein NETWORKED 4B {ECO:0000303\|PubMed:22840520} |  |
| VILI5_ARATH | VILI5_ARATH Villin-5 {ECO:0000303\|PubMed:20807879} |  |
| MAD16_ORYSJ | MAD16_ORYSJ MADS-box transcription factor 16 |  |
| MYST1_ARATH | MYST1_ARATH Histone acetyltransferase of the MYST family 1 |  |
| PRS4A_ARATH | PRS4A_ARATH 26S proteasome regulatory subunit 4 homolog A |  |
| YUC6_ARATH | YUC6_ARATH Indole-3-pyruvate monooxygenase YUCCA6 |  |
| RBL2_ARATH | RBL2_ARATH RHOMBOID-like protein 2 {ECO:0000303\|PubMed:16223493, ECO:0000303\|PubMed:17181860} |  |
| ACA10_ARATH | ACA10_ARATH Calcium-transporting ATPase 10, plasma membrane-type |  |
| ACCR4_ARATH | ACCR4_ARATH Serine/threonine-protein kinase-like protein CCR4 |  |
| BH034_ARATH | BH034_ARATH Transcription factor bHLH34 | bHLH |
| C7101_ARATH | C7101_ARATH Cytochrome P450 710A1 {ECO:0000303\|PubMed:16531502} |  |
| C85A_PHAVU | C85A_PHAVU Cytochrome P450 85A |  |
| CCAMK_LILLO | CCAMK_LILLO Calcium and calcium/calmodulin-dependent serine/threonine-protein kinase |  |
| CIPK5_ARATH | CIPK5_ARATH CBL-interacting serine/threonine-protein kinase 5 |  |
| CRK22_ARATH | CRK22_ARATH Cysteine-rich receptor-like protein kinase 22 | GeBP |
| ERF12_ARATH | ERF12_ARATH Ethylene-responsive transcription factor ERF012 | ERF |
| GAT20_ORYSJ | GAT20_ORYSJ GATA transcription factor 20 {ECO:0000303\|PubMed:15084732} |  |
| GAT28_ARATH | GAT28_ARATH GATA transcription factor 28 |  |
| HFB2B_ARATH | HFB2B_ARATH Heat stress transcription factor B-2b |  |
| HFB2C_ORYSJ | HFB2C_ORYSJ Heat stress transcription factor B-2c |  |
| INP1_ARATH | INP1_ARATH Protein INAPERTURATE POLLEN1 |  |
| MEB1_ARATH | MEB1_ARATH Membrane protein of ER body 1 |  |
| MSL1_ORYSJ | MSL1_ORYSJ Leucine-rich repeat receptor protein kinase MSL1 {ECO:0000305} |  |
| MYC4_ARATH | MYC4_ARATH Transcription factor MYC4 |  |
| PMA8_ARATH | PMA8_ARATH ATPase 8, plasma membrane-type |  |
| ROGFC_ARATH | ROGFC_ARATH Rop guanine nucleotide exchange factor 12 |  |
| SCAM1_ORYSI | SCAM1_ORYSI Putative secretory carrier-associated membrane protein 1 |  |
| SCAM1_ORYSJ | SCAM1_ORYSJ Secretory carrier-associated membrane protein 1 |  |
| SCY1_DICDI | SCY1_DICDI Probable inactive serine/threonine-protein kinase scy1 |  |
| SMG1_HUMAN | SMG1_HUMAN Serine/threonine-protein kinase SMG1 |  |
| STK16_RAT | STK16_RAT Serine/threonine-protein kinase 16 |  |
| T214A_XENLA | T214A_XENLA Transmembrane protein 214-A |  |
| TF3C6_HUMAN | TF3C6_HUMAN General transcription factor 3C polypeptide 6 |  |
| TF3C6_MOUSE | TF3C6_MOUSE General transcription factor 3C polypeptide 6 |  |
| TGA6_ARATH | TGA6_ARATH Transcription factor TGA6 |  |
| TM87B_HUMAN | TM87B_HUMAN Transmembrane protein 87B |  |
| TMM56_HUMAN | TMM56_HUMAN Transmembrane protein 56 |  |
| WAKLF_ARATH | WAKLF_ARATH Wall-associated receptor kinase-like 6 |  |
| WAKLI_ARATH | WAKLI_ARATH Wall-associated receptor kinase-like 22 |  |
| WRK53_ARATH | WRK53_ARATH Probable WRKY transcription factor 53 | WRKY |
| WRKY3_ARATH | WRKY3_ARATH Probable WRKY transcription factor 3 | WRKY |
| Y2685_ARATH | Y2685_ARATH Probably inactive receptor-like protein kinase At2g46850 |  |
| ERF5_NICSY | ERF5_NICSY Ethylene-responsive transcription factor 5 | ERF |
| GATA4_ARATH | GATA4_ARATH GATA transcription factor 4 |  |
| IRE1_CAEEL | IRE1_CAEEL Serine/threonine-protein kinase/endoribonuclease ire-1 |  |
| NQR_ARATH | NQR_ARATH NADPH:quinone oxidoreductase |  |
| SAPK6_ORYSJ | SAPK6_ORYSJ Serine/threonine-protein kinase SAPK6 |  |
| TMM60_MOUSE | TMM60_MOUSE Transmembrane protein 60 |  |
| TPRA1_CHICK | TPRA1_CHICK Transmembrane protein adipocyte-associated 1 homolog |  |
| VEMP_CVEMC | VEMP_CVEMC Envelope small membrane protein |  |
| Y1864_ARATH | Y1864_ARATH Probable receptor-like protein kinase At1g80640 |  |
| CAC1A_APIME | CAC1A_APIME Voltage-dependent calcium channel type A subunit alpha-1 |  |
| CAC1A_DROME | CAC1A_DROME Voltage-dependent calcium channel type A subunit alpha-1 |  |
| CML11_ORYSJ | CML11_ORYSJ Probable calcium-binding protein CML11 |  |
| MA652_ARATH | MA652_ARATH 65-kDa microtubule-associated protein 2 |  |
| U73C1_ARATH | U73C1_ARATH UDP-glycosyltransferase 73C1 |  |
| UBIQ1_CAEEL | UBIQ1_CAEEL Polyubiquitin-A |  |
| UBP20_ARATH | UBP20_ARATH Ubiquitin carboxyl-terminal hydrolase 20 |  |
| UBP21_ARATH | UBP21_ARATH Ubiquitin carboxyl-terminal hydrolase 21 |  |
| UPL2_ARATH | UPL2_ARATH E3 ubiquitin-protein ligase UPL2 |  |
| ENGB_BACMF | ENGB_BACMF Probable GTP-binding protein EngB |  |
| NSRB_ARATH | NSRB_ARATH Nuclear speckle RNA-binding protein B |  |

**Table S11.** Primer sequences used in this study.

| **Primer** | **Sequence** |
| --- | --- |
| ActinRrefqPCRFw1 | GTTGCACCACCAGAGAGGAA |
| ActinRrefqPCRRv1 | TGACTCGTCGTACTCCCCTT |
| ActinDtorqPCRFw1 | AGCTCGCATATGTGGCTCTT |
| ActinDtorqPCRRv1 | ACCATCAGGCAGCTCGTAAC |
| ActinFbraqPCRFw2 | GCCATCCTTCGTTTGGACCT |
| ActinFbraqPCRRv1 | ACAATTTCCCGCTCAGCAGT |
| ActinEcaqPCRFw2 | TTACAATGAGCTTCGTGTTGC |
| ActinEcaqPCRRv2 | CCCAGCACAATACCTGTAGTAC |
| EcaINP1Fw | ATGATCAAAGCTGCAGCTCGA |
| EcaINP1Rv | AATGCCTGATAATGGAATCTTGC |
| INP1FbraqPCRFw2 | TCGAACAGTGCCAGATTCCC |
| INP1FbraqPCRRv2 | CAGTCGCAGAAAGCCCCATA |
| INP1DtorqPCRFw3 | CCTACTCTCGTTACCCGTGC |
| INP1DtorqPCRRv3 | GCCTTTGATACCTCCTGCCT |
| INP1RrefqPCRFw3 | TCCCAACTTGGCACAACTCA |
| INP1RrefqPCRRv3 | TCATCGTCGTCGTAATTGAGTGA |
| DYT_FbraFw2 | TCAGTTTCAGAGGAGCAACCC |
| DYT_FbraRv2 | AGCAAGAAGTTCCTCACCTGT |
| DYT_RrefFw1 | GCCACTCTTGACGACGCTAT |
| DYT_RrefRv1 | CTTCAACGGCTCGAGGAACT |
| DYT_DtorFw1 | GGAACCGACCAGCTGAATGAT |
| DYT_DtorRv1 | ATCCAAGAACAAGCCAGCACA |
| MAD16_DtorFw2 | TGCTATCGTAAGCCTGGTGG |
| MAD16_DtorRv2 | TGCGTGCGAGGTTTTTACAC |
| MAD16_FbraFw2 | TGCACTAGCAAACGAAGGGG |
| MAD16_FbraRv2 | AAGATTGGGCTGACTTGGCT |
| MAD16_RrefFw2 | CATGTTCTGCACTCGCACAC |
| MAD16_RrefRv2 | AGCCGCATAAACTGCACGTA |
| MAD16_EcaFw2 | GAAATGGCGAATGGTGGTGTT |
| MAD16_EcaRv2 | AAGCAAGGCGTAGATCGTGA |
| AMS_DtorFw1 | AAGCTGCCAGTACTCACGTC |
| AMS_DtorRv1 | GGGTCGGCCATACGAAAGAA |
| AMS_EcaFw2 | TGCGCGAACGTGATTCTTTC |
| AMS_EcaRv2 | CTCCAGAAATTCCCCTGTCCC |
| AMS_RrefFw1 | TGCTGCAAAACATGTACGCC |
| AMS_RrefRv1 | AGCATTACGCCATCCATCCA |
| AMS_FbraFw1 | GCCATGGGTTATCTCGGACT |
| AMS_FbraRv1 | ACATCACTTGTTTGAGGAGGT |
| Fbra_VILI5_Fw | GCTCTTCTTGGACACCGGAA |
| Fbra_VILI5_Rv | TGGTTGTTCACGCTACCAGA |
| Fbra_1310_Fw | ACCACTGGTAAAGGTGGCAA |
| Fbra_1310_Rv | ATTCCCTGAACTAGCCGGTG |
| Fbra_BBD1_Fw | GGATTACGCCCCACATCCTG |
| Fbra_BBD1_Rv | GCACAGCAGCCATGAGTAAC |
| Fbra_NET4b_Fw | TGATAAAAGTTGTCACCCAGACA |
| Fbra_NET4b_Rv | CGGGCGGCTCTATCAATGAC |
| Fbra_ASD2_Fw | GTCATGGTTGCAGGAAATGCT |
| Fbra_ASD2_Rv | GTGTCCCATTGCTGCTCGAA |
| Fbra_WTR8_Fw | AGCCAAGCTTTGGGGAATCA |
| Fbra_WTR8_Rv | ACCCGGTAGCTCGTTAGAGT |
| Rref_VILI5_Fw | GCAGCTCCCAACCCTTACAA |
| Rref_VILI5_Rv | TGTCGTCGACCGAAGAACTC |
| Rref_1310_Fw | TGCGAAACTCCTCCAGACAA |
| Rref_1310_Rv | TCACAAGTGGACGTGCATGA |
| Rref_BBD1_Fw | AGCACTAAGGGTTCCATGCC |
| Rref_BBD1_Rv | GAACATGGCTGGGCTGATCT |
| Rref_NET4b_Fw | GGAACCGCGAGATGGAAGAT |
| Rref_NET4b_Rv | ACAAGCACAGCACTGACGTA |
| Rref_ASD2_Fw | CGCACATTGCTTCTTGTGAGT |
| Rref_ASD2_Rv | ATGCAAGCCGGTTCATGGTA |
| Rref_WTR8_Fw | AGAGGACCCGTTTTCGTGAC |
| Rref_WTR8_Rv | GCACCAATGACACTTCCCAG |
| Eca_VILI5_Fw | AGACAAGTCACAGCGAACCC |
| Eca_VILI5_Rv | AGCAGCTAGTGCAGTGAAGG |
| Eca_1310_Fw | AGTTGGCAACGAAGTGATTCC |
| Eca_1310_Rv | AACCCCCTTTGCTTGAGAGC |
| Eca_BBD1_Fw | TTCAGGAACTACGGCTCATTCT |
| Eca_BBD1_Rv | CTCATTGCAGCTGGGTGGTA |
| Eca_NET4b_Fw | CACTCCTGATCGAAGGCCAA |
| Eca_NET4b_Rv | GACACTAGTGCTGCCTCCAG |
| Eca_ASD2_Fw | AGTGACTCCGTCGTCCAGTA |
| Eca_ASD2_Rv | GGGTCGCCTTGACCTAGAAG |
| Eca_WTR8_Fw | CATGTTTAACCCCCTCGGGA |
| Eca_WTR8_Rv | TCCTCCAATGCAACTTCCCA |
| Dtor_VILI5_Fw | AGCAAGCTTTGACTATTGGTGA |
| Dtor_VILI5_Rv | GAGGCTCGAACCCTTCCATT |
| Dtor_1310_Fw | CCTTTGGAAGGCCAACCAGT |
| Dtor_1310_Rv | GCTCAGATCGATGCTGTCCA |
| Dtor_BBD1_Fw | GGTCAACCGTGTGTTGAAGC |
| Dtor_BBD1_Rv | GGGTAAGCTTGTCTCGCCAT |
| Dtor_NET4b_Fw | GGGGTGTGTTCACCCGTTTA |
| Dtor_NET4b_Rv | TGACCCAAAAAGACCCCCAC |
| Dtor_ASD2_Fw | GGCACAGTCTTCGCTTTGTT |
| Dtor_ASD2_Rv | TGTCAAGCATTTCCCTCCCTT |
| Dtor_WTR8_Fw | CCTTCAACGGCCAAGCAAAG |
| Dtor_WTR8_Rv | TCGAACGGTGGTGATCCTTG |
| ELMOD_A_Fbra_Fw | TTTGGGGAGTTGAATTGGCG |
| ELMOD_A_Fbra_Rv | CTTCGTGGTGCCAGAACAAA |
| ELMOD_B_Fbra_Fw | AGGGGTGGTGGCTTCATATC |
| ELMOD_B_Fbra_Rv | ATAGCCCTATCGCCTTCCTG |
| ELMOD_E_Fbra_Fw | GGACTCTGGAGCTCTTGGTT |
| ELMOD_E_Fbra_Rv | CCTGGGGAACTGCGTATACA |
| ELMOD_A_Rref_Fw | AAGCTATGGATGGACGTGGT |
| ELMOD_A_Rref_Rv | CCTTTTCCAATCCATGCCCC |
| ELMOD_B_Rref_Fw | TTCAGGGGTGGTGGTTTCAT |
| ELMOD_B_Rref_Rv | CGATCACCTTCCTGCTTTCG |
| ELMOD_E_Rref_Fw | GAGAGCCCCATTTCTGACCT |
| ELMOD_E_Rref_Rv | GGTGGTGAGACTGGGATTGA |
| ELMOD_A_Dtor_Fw | ACAGACGGCTTGAAGAAGGA |
| ELMOD_A_Dtor_Rv | TGAAGCCCTCAACGATCACT |
| ELMOD_B_Dtor_Fw | TGATGGTTGCAATCCTGAGC |
| ELMOD_B_Dtor_Rv | CTTGCCAGCCCATCTCTTTC |
| ELMOD_E_Dtor_Fw | GTCTGGACACTGGAGCTCTT |
| ELMOD_E_Dtor_Rv | AAGCAGGCATCAAACGACTG |
| ELMOD_A_Ecal_Fw | tgatttatccccgtccgagg |
| ELMOD_A_Ecal_Rv | ttagcgcttcctggtgatct |
| ELMOD_B_Ecal_Fw | aggaaggtgatagggcgatg |
| ELMOD_B_Ecal_Rv | aacgtccttggtttgactgc |
| ELMOD_E_Ecal_Fw | tgattttaggggctgtgggt |
| ELMOD_E_Ecal_Rv | cccaaattgccctcttaccg |
